# Dynamics of actively dividing prokaryotes in the western Mediterranean Sea

**DOI:** 10.1038/s41598-022-06120-y

**Published:** 2022-02-08

**Authors:** Catalina Mena, Patricia Reglero, Rosa Balbín, Melissa Martín, Rocío Santiago, Eva Sintes

**Affiliations:** 1 Instituto Español de Oceanografía, Centre Oceanogràfic de Les Balears, Ecosystem Oceanography Group (GRECO), Palma, Spain; 2 Present Address: IFREMER – Centre Bretagne Z.I. Technopôle Brest-Iroise Pointe du Diable, BP70, 29280 Plouzané, France

**Keywords:** Microbial ecology, Microbial communities, Environmental microbiology, Marine microbiology

## Abstract

Microbial community metabolism and functionality play a key role modulating global biogeochemical processes. However, the metabolic activities and contribution of actively growing prokaryotes to ecosystem energy fluxes remain underexplored. Here we describe the temporal and spatial dynamics of active prokaryotes in the different water masses of the Mediterranean Sea using a combination of bromodeoxyuridine labelling and 16S rRNA gene Illumina sequencing. Bulk and actively dividing prokaryotic communities were drastically different and depth stratified. Alteromonadales were rare in bulk communities (contributing 0.1% on average) but dominated the actively dividing community throughout the overall water column (28% on average). Moreover, temporal variability of actively dividing Alteromonadales oligotypes was evinced. SAR86, Actinomarinales and Rhodobacterales contributed on average 3–3.4% each to the bulk and 11, 8.4 and 8.5% to the actively dividing communities in the epipelagic zone, respectively. SAR11 and Nitrosopumilales contributed less to the actively dividing than to the bulk communities during all the study period. Noticeably, the large contribution of these two taxa to the total prokaryotic communities (23% SAR11 and 26% Nitrosopumilales), especially in the meso- and bathypelagic zones, results in important contributions to actively dividing communities (11% SAR11 and 12% Nitrosopumilales). The intense temporal and spatial variability of actively dividing communities revealed in this study strengthen the view of a highly dynamic deep ocean. Our results suggest that some rare or low abundant phylotypes from surface layers down to the deep sea can disproportionally contribute to the activity of the prokaryotic communities, exhibiting a more dynamic response to environmental changes than other abundant phylotypes, emphasizing the role they might have in community metabolism and biogeochemical processes.

## Introduction

Microbes are the major biological drivers of ocean biogeochemical cycles^1^. The major redox reactions key to the global biogeochemical processes are carried out by highly diverse prokaryotic taxa^2–5^. Microbial communities are dominated by few abundant taxa and by a large number of low abundant or rare prokaryotes^6^, that provide a seed bank of organisms able to grow and become dominant when the conditions change^7,8^. However, the contribution of specific taxa to the element cycles and energy flows depends not only on their abundance but also on their activity and growth rates^9^. Rare phylotypes with high growth rates can contribute significantly to community activity^10^. Besides, changes in the activity of dominant taxa can also alter nutrient fluxes and ecosystem production^11^. Yet, the growth and metabolic activities of specific taxa in natural environments, particularly in the dark ocean, remain largely unknown.

The inactive or slow-growing cells exhibit different adaptations such as genomic streamlining^12^, and/or resistance mechanisms against predation and viral lysis^13^. These slow-growing cells are more likely to be oligotrophs adapted to low nutrient supply, such as the abundant SAR11 clade. Although the ecological strategy of this clade is in debate^14,15^, recent findings suggest that the success of some SAR11 ecotypes could be related to low grazing and viral losses and to their higher competitiveness in limited resource environments^15,16^. Archaea have been reported to have a significant role in the metabolism of dark ocean communities due to their high relative abundances^17,18^ despite exhibiting low growth rates compared to bacteria.

The more active or fast-growing cells are often related to opportunistic strategies and copiotrophic metabolism^19^. Their higher nutrient requirements and the ability to take advantage of sporadic increased energy supply results in a feast or famine response, involving variable growth rates and changes in the contribution to community abundance^10^. Alteromonadales, Vibrionales and Rhodobacterales, related to opportunistic behaviours, are common active and fast-growing groups in ocean environments^2,20,21^. Cells allocating fewer or no resources to resistance machinery can grow faster, however, actively dividing cells are preferentially infected by viruses^22^ and grazing rates are higher on the larger and more nutritive fast-growing cells than in the smaller and starved slow-growing cells^23^. Consequently, fast-growing cells less likely yield high abundances^9^.

In previous studies, we have characterized the spatial and temporal patterns of bulk prokaryotic communities in the sunlit and dark Mediterranean open sea^24,25^. The aim of this study was to enhance our knowledge and characterize the temporal and spatial dynamics of total vs. actively dividing prokaryotic communities from surface down to the deep Mediterranean Sea waters. To discriminate the actively dividing phylotypes from the bulk community, bromodeoxyuridine (BrdU) labelling and immunocapturing was used in combination with Illumina sequencing of 16S rRNA gene. BrdU is an analogue of thymidine that is incorporated into the newly synthetized DNA, used as a nonradioactive alternative to measure cell growth^26^. Moreover, this technique together with 16S rRNA gene sequencing permits to assess the contribution of rare and abundant taxa to the actively dividing communities at a detailed taxonomic level. Other nonradioactive methods used to characterize active prokaryotes are based on labelling of compounds that incorporate into proteins or DNA or on dye binding^27–29^. These techniques have been combined with fluorescence in situ hybridization techniques^27^, allowing only a limited taxonomic characterization, whereas detailed taxonomy can only be obtained after combination with sorting^30^, and thus require specialized instrumentation. Our results evidence the remarkable contribution and dynamism of the rare and disproportionately actively dividing phylotypes and their temporal variability within the overall water column, suggesting they have a crucial role in the biogeochemical processes.

## Results

### Environmental conditions

The depth layers studied differed in physical and chemical characteristics. Three contrasting seasons were characterized: winter, summer and autumn, corresponding to the three sampling cruises. Surface and deep chlorophyll maximum (DCM), i.e., epipelagic zone, exhibited more pronounced environmental variability over the seasons than the dark ocean (Supplementary Fig. S1 and S2).

In winter the water column was well-mixed, with temperature ranging between 13.1 and 14.8 °C. Maximum chlorophyll-*a* (Chl-*a*) concentration was detected at 0–30 m depth, ranging between 0.75 and 0.97 mg m^−3^. Nitrate, nitrite and phosphate in winter ranged between 0.04–4.46, 0–0.20 and 0.01–0.14 µM in the upper 100 m, respectively. Stratification of the upper water column was apparent during summer, when temperature decreased from 22.8–25.9 °C at surface to < 14 °C below 100 m. Surface Chl-*a* (< 0.05 mg m^−3^) and inorganic nutrients concentrations were remarkably low (nitrate, nitrite and phosphate concentrations were below the detection limit). The DCM was located at 66–98 m, reaching 0.64–1.34 mg m^−3^. Nitrate, nitrite and phosphate concentrations at the DCM ranged between 1.77–3.13, 0–0.26 and 0.01–0.09 µM, respectively. During autumn, the thermocline structure was disrupted (Supplementary Fig. S1). Chl-*a* concentrations were intermediate between the stratified and mixed water column conditions. Nitrate, nitrite and phosphate were negligible at surface and increased below 50 m. Silicate concentration values did not show notable differences between seasons in the epipelagic layer, ranging between 0.38 and 4.10 µM within the first 100 m during the study period (Supplementary Fig. S2).

The Levantine intermediate water (LIW, corresponding to the mesopelagic layer) core depth ranged between 334 and 500 m during the sampling period and was characterized by a salinity maximum (38.56–38.60 PSU) and oxygen minimum (165.5–184.9 µmol kg^−1^) as compared to the other water masses. At the bathypelagic, including samples from old western Mediterranean deep water (oWMDW, sampled at 1,000 m) and bottom water (1,370–2,560 m), salinity decreased to 38.48 (PSU). Oxygen concentration increased from 180.6–189.4 µmol kg^−1^ at oWMDW to 193.9–197.4 µmol kg^−1^ at the bottom. Below the photic zone (> 200 m), the concentration of nitrate and phosphate increased, peaking at the LIW (8.58–9.87 and 0.35–0.43 µM for nitrate and phosphate, respectively) (Supplementary Fig. S2). Silicate concentrations increased with depth and from winter (7.24–8.80 µM at > 1,000 m) to autumn (8.13–10.74 µM at > 1,000 m).

### Abundance and diversity of prokaryotic communities

Prokaryotic abundances in winter were higher at surface (7.6 ± 0.9 × 10^5^ cells mL^−1^, mean ± s.e.m.), decreasing exponentially with depth down to 0.8 ± 0.1 × 10^5^ cells mL^−1^ at the bottom (Supplementary Fig. S3). High nucleic acid (HNA) cells contributed 42 ± 4% at surface, increased to 49 ± 3% at 50 m-LIW depths and decreased to 44 ± 4% at waters below the LIW (Supplementary Fig. S3). Maximum abundance in summer was located at 25–75 m depth (7.2 ± 1.6 × 10^5^ cells mL^−1^), and the minimum abundance at the bottom waters (0.7 ± 0.1 × 10^5^ cells mL^−1^) (Supplementary Fig S3). At surface, 35 ± 3% of the cells were HNA content in summer, increasing its contribution with depth up to 55 ± 2% at the bottom (Supplementary Fig. S3). In autumn, prokaryotic abundance decreased from surface (7.2 ± 0.3 × 10^5^ cells mL^−1^) to bottom waters (0.5 ± 0.04 × 10^5^ cells mL^−1^), whereas HNA cells contribution increased from surface (41 ± 4%) to bottom waters (58 ± 2%) (Supplementary Fig. S3).

The 16S rRNA sequences clustered in a total of 12,015 different ASVs before rarefaction. Total and actively dividing communities harboured 22 and 51% unique ASVs, respectively, whereas they shared 26% of all ASVs. 4.3% of all ASVs were shared by all depth layers, 11 and 8% were unique to surface and DCM, respectively, whereas 18, 16 and 15% of ASVs were unique to LIW, oWMDW and bottom communities, respectively.

Actively dividing communities (BrdU-labelled) were phylogenetically more diverse than total communities at all depths (ANOVA, *P* < 0.01) and their diversity increased with depth (Supplementary Fig. S4). Actively dividing communities showed higher Shannon diversity and Pielou’s evenness at surface (ANOVA, *P* < 0.05) as compared to total communities. However, at the meso- and bathypelagic water masses (LIW, oWMDW, bottom), higher Shannon diversity and Pielou’s evenness was measured in total than in actively dividing communities (ANOVA, *P* < 0.05) (Supplementary Fig. S4).

### Total and actively dividing community structure

**Figure 1:**
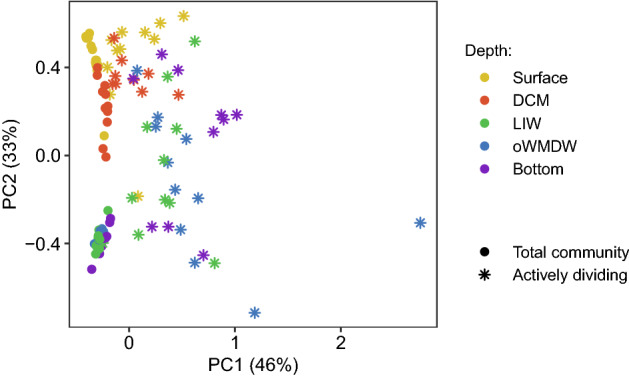
Principal Coordinates Analysis (PCoA) of prokaryotic communities based on weighted UniFrac distances. The variance explained is shown for each axis. Colours indicate depth and shapes indicate total versus actively dividing communities. DCM: deep chlorophyll maximum; LIW: Levantine intermediate water; oWMDW: old western Mediterranean deep water.

Prokaryotic communities from the epipelagic layer (surface and DCM) and the meso- (LIW) and bathypelagic (oWMDW and bottom) layers were drastically different, as revealed by principal coordinate analysis based on weighted UniFrac distances (Fig. 1). Moreover, actively dividing differed from total communities (Fig. 1). Depth layer and activity (i.e., total vs. actively dividing communities) explained 28 and 26% of the overall community variability, respectively (Supplementary Table S1). Besides, season also influenced the community composition explaining 2% of variability (Supplementary Table S1). Actively dividing communities exhibited a larger variability, however, less clear clustering according to depth layers as compared to total communities (Fig. 1). Similar results were obtained using Bray Curtis, Jaccard and unweighted UniFrac distance matrices, however, the fraction of the community variability explained was smaller (Supplementary Fig. S5 and Table S1). Consequently, we used weighted UniFrac distances matrices for further analysis. 49 and 52% of the variability of surface and DCM communities, respectively, was explained by activity and season (Supplementary Table S2), activity explaining a higher proportion of the variability than season at both depths (30 and 42% at surface and DCM communities, respectively) (Supplementary Table S2).

The difference between total and actively dividing communities accounted for a higher proportion of the variability of the meso- and bathypelagic water masses communities as compared to the surface community, explaining 49, 36 and 61% of community variability of the LIW, oWMDW and bottom waters, respectively (Supplementary Table S2). LIW actively dividing communities were influenced by season (10%); however, season was not significant neither for LIW total communities nor for oWMDW and bottom water total and actively dividing communities (Supplementary Table S2).

### Epipelagic zone total and actively dividing prokaryotic communities

**Figure 2:**
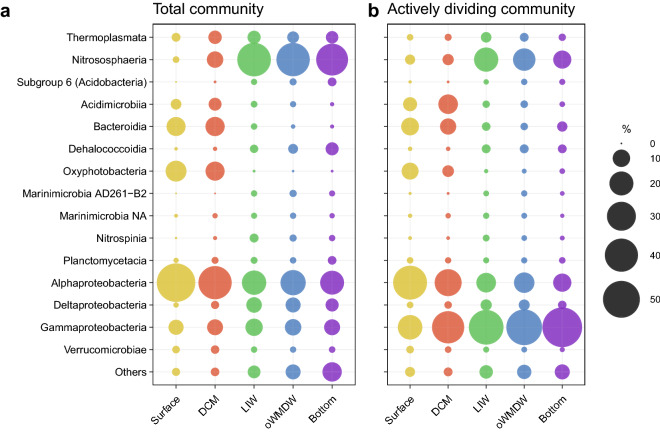
Contribution of phylotypes to (**a**) total and (**b**) actively dividing communities in different depth layers. Bubbles show the relative abundance of taxa at the Class level. Taxa contributing ≤ 0.5% of abundance in total and/or actively dividing communities are combined in ‘Others’ group. DCM: deep chlorophyll maximum; LIW: Levantine intermediate water; oWMDW: old western Mediterranean deep water; NA: unidentified.

The most abundant classes of surface and DCM communities were Nitrososphaeria, Acidimicrobiia, Bacteroidia, Oxyphotobacteria, Alpha- and Gammaproteobacteria, both in total and actively dividing communities (Fig. 2). The relative abundance of Acidimicrobiia and Gammaproteobacteria increased from 3 and 7% in total communities to 6 and 21% in actively dividing communities at the surface waters, respectively. Similarly, these two classes increased their contribution from 5 and 8% in total communities to 13 and 37% in actively dividing communities, respectively, at the DCM. Nitrososphaeria relative abundance increased from 1 to 3.6% in total and actively dividing surface communities, respectively. On the contrary, Bacteroidia, Oxyphotobacteria and Alphaproteobacteria decreased their contribution from 12, 15 and 55% in total communities to 10, 9, and 42% in actively dividing communities at the surface waters, respectively; and from 12, 12 and 40% in total communities to 8.5, 4 and 26% in actively dividing communities at the DCM, respectively. Nitrososphaeria relative abundance decreased from 9 to 3.6% in total and actively dividing DCM communities, respectively (Fig. 2).

Total community composition spatial and temporal dynamics has been previously reported^24^. Here, we briefly describe the main features observed over the studied period, in order to facilitate comparison to the actively dividing community dynamics. Seasonality of epipelagic communities was more pronounced at surface waters than at DCM. Family *Cyanobiaceae* showed its minimum contribution to the surface water community in summer, accounting for 15 ± 6 (mean ± s.e.m.), 9 ± 3 and 21 ± 1% in winter, summer and autumn, respectively. Members of this family contributed from 4 to 19% throughout the study at DCM communities (Supplementary Fig. S6) with no clear seasonal trend. Contrarily, SAR116 and AEGEAN-169 from Rhodospirillales increased their contribution to surface water communities in summer, accounting for 10 ± 0.6 and 5 ± 1%, respectively, compared to 3 ± 0.4 and 2 ± 0.2% of contribution in the other seasons (Supplementary Fig. S6). Other abundant phylotypes at the family level were *Flavobacteriaceae* (contributing 4–15% throughout the study), SAR11 Clade I (20–47% at surface and 16–29% at DCM), Clade II (3–8% throughout the study), *Rhodobacteraceae* (1.4–4% throughout the study), SAR86 (accounting 1.4–5.4%) and *Actinomarinaceae* (contributing 1–6.5%). *Nitrosopumilaceae* was more abundant in winter (1.6–13%) than in other seasons (< 1%) at surface waters, whereas at the DCM exhibited maxima contributions in summer (6–19%). Marine Group II archaea comprised 0.2–9% of the surface community in winter and autumn and of the DCM communities; however, this archaeal group was undetectable in summer surface waters (Supplementary Fig. S6).

Actively dividing communities were characterized by a large variability of phylotypes contribution throughout the study period (Supplementary Fig. S6). Alteromonadales contribution to actively dividing communities remarkably increased in summer, ranging from 2% to up to 22% at surface and 76% at DCM. SAR86 families also increased their contribution to the actively dividing communities in summer, contributing 3 ± 1, 11 ± 6 and 8 ± 1% at surface and 8 ± 5, 8 ± 6 and 4 ± 2% at DCM in winter, summer and autumn, respectively. *Actinomarinaceae* and *Rhodobacteraceae* accounted for 4–28 and 3–15% of actively dividing communities in winter, respectively, increasing their contribution to actively dividing communities in summer, ranging between 0.4–37 and 0.4–22%, respectively, and subsequently decreased in autumn, ranging between 3–21 and 5–14%, respectively. *Cyanobiaceae* had a lower contribution to actively dividing communities than to total communities at both surface (accounting 1.4–18%) and DCM (0.3–14%). Phylotypes with different contribution at surface and DCM active communities were SAR11 Clade I, SAR116 and AEGEAN-169 from Rhodospirillales, contributing 7–26, 0.4–12 and 1–10% at surface and 0.2–12, 0–2 and 0.3–5% at DCM, respectively. Marine Group II and *Nitrosopumilaceae* accounted for 0–3% and 0–8% of the actively dividing communities, respectively, with the exception of St E in summer at surface (25%) (Supplementary Fig. S6).

### Meso- and bathypelagic zones total and actively dividing prokaryotic communities

The meso- (corresponding to LIW) and bathypelagic (including oWMDW and bottom waters) layers total and actively dividing communities were dominated by Thermoplasmata, Nitrososphaeria, Dehalococcoidia, Alpha-, Delta- and Gammaproteobacteria (Fig. 2). Bacteroidia and Gammaproteobacteria increased remarkably their contribution from 0.2–0.9 and 8–10% in total communities to 1.3–3 and 44–58% in actively dividing meso- and bathypelagic communities, respectively. Similar to the epipelagic layer communities, Thermoplasmata, Nitrososphaeria and Alphaproteobacteria decreased their contribution from 4–5, 37–41 and 20–22% in total communities to 1–3, 11–20, and 11–14% in actively dividing meso- and bathypelagic communities, respectively. Besides, Deltaproteobacteria decreased its contribution from 5–7.7 to 2–3.6% in total and actively dividing communities (Fig. 2).

Total community composition from the meso- and bathypelagic water masses remained similar throughout the different seasons and stations (Supplementary Fig. S6). The most abundant phylotypes at family level were *Nitrosopumilaceae* (28–54%), SAR11 Clade I (1.6–12.7%) and II (3–11.4%), families from SAR406 clade (3–7.4%) and SAR324 clade (2–10.6%). Families from Marine Group II (0.5–7%), from SAR202 (1–7%), *Nitrospinaceae* (0.5–4%) and families from HOC36 (0.4–2%) and UBA10353 (1.2–3.7%) (Gammaproteobacteria) also contributed considerably to total communities (Supplementary Fig. S6).

Contrarily, the composition of meso- and bathypelagic actively dividing communities largely varied (Supplementary Fig. S6). Alteromonadales phylotypes drastically changed their contribution among actively dividing communities, reaching > 15% of contribution in more than 70% of the samples. In particular, *Alteromonadaceae* and *Idiomarinaceae* contributed up to 76 and 74% to the actively dividing communities, respectively. Major increases of *Alteromonadaceae* were observed in summer, whereas *Idiomarinaceae* were observed mainly in winter at the three depths (Supplementary Fig. S6). Other families from Alteromonadales that separately contributed < 0.5%, together comprised up to 32% of the actively dividing community (Supplementary Fig. S6). *Nitrosopumilaceae*, SAR11 Clade I, SAR11 Clade II and SAR324 comprised 3–40, 0.6–7.6, 0.6–8 and 0.2–6% of the actively dividing communities, respectively. Phylotypes putatively assigned to SAR406 clade contributed between 0.5 and 8.7% to actively dividing communities.

### Depth specific actively dividing phylotypes

**Figure 3:**
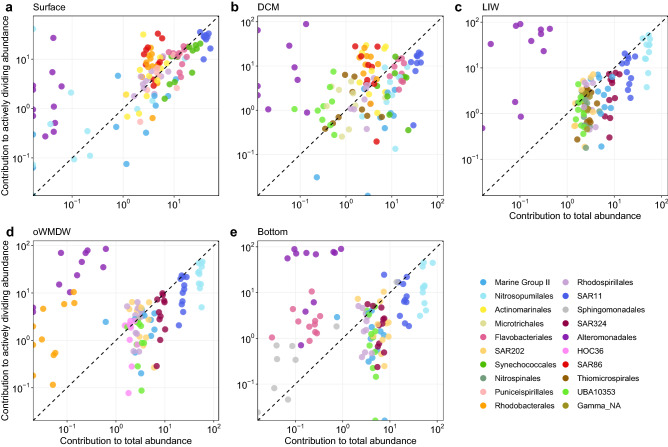
Relative contribution of phylotypes to total vs. actively dividing communities. Phylotypes at the Order level with > 1.5% of abundance are plotted for (**a**) surface, (**b**) deep chlorophyll maximum (DCM), (**c**) Levantine intermediate water (LIW), (**d**) old western Mediterranean deep water (oWMDW) and (**e**) bottom. Dashed lines indicate equal contribution to total and actively dividing communities, phylotypes above the line contribute more to the actively dividing community than to the total community. NA: unidentified.

The differential contribution of specific phylotypes to actively dividing as compared to total communities changed with depth. Alteromonadales contributed more to actively dividing than total communities in all depth layers (Fig. 3). On the contrary, Marine Group II and SAR11 contributed more to total than to actively dividing communities in all depths (Fig. 3). Actinomarinales, Rhodobacterales and SAR86 contributed more to actively dividing than to total communities in surface and DCM waters (Fig. 3a, b). UBA10353 (Gammaproteobacteria) at the DCM also differentially contributed more to the actively dividing than to total communities (Fig. 3b). Synechococcales contribution were lower to actively dividing than to total communities, both at surface and DCM (Fig. 3a, b). Nitrosopumilales, SAR324 and UBA10353 (Gammaproteobacteria) contributed more to total than to actively dividing communities of LIW, oWMDW and bottom waters (Fig. 3c–e). Several of the most abundant phylotypes (> 1.5% of relative abundance) contributed similarly to total and actively dividing communities, e.g., Microtrichales at the DCM and Rhodospirillales and SAR202 at the LIW (Fig. 3b, c). Noteworthy, Flavobacteriales and Sphingomonadales showed a high increase in its contribution to actively dividing communities in bottom waters (Fig. 3e).

**Figure 4:**
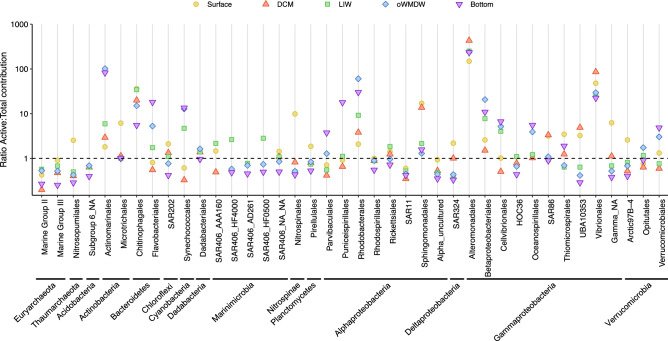
Actively dividing to total contribution ratio of phylotypes. Phylotypes at the Order level with ≥ 0.6% of abundance in at least one water mass are plotted. The different depth layers are depicted in different shapes and colours. Corresponding Phyla and Class (for Proteobacteria), are specified. Dashed line indicates ratio 1. DCM: deep chlorophyll maximum; LIW: Levantine intermediate water; oWMDW: old western Mediterranean deep water; NA: unidentified.

Although some phylotypes contributed more to actively dividing than to total communities throughout all depth layers, their differential relative contribution varied with depth. The ratio between the contribution to actively dividing vs. contribution to total communities of Actinomarinales, Rhodobacterales and Betaproteobacteriales increased with depth; whereas Chitinophagales, Sphingomonadales and Vibrionales ratios were larger at the upper layers and decreased with depth (Fig. 4). Unexpectedly, some phylotypes significantly more abundant in surface or DCM waters, such as Flavobacteriales, Synechococcales, Parvibaculales, Puniceispirillales and Cellvibrionales, were dividing relatively slow (ratio of contribution to active vs. total < 1) in surface and DCM waters and showed a larger ratio of contribution to actively dividing versus total communities in the meso- and bathypelagic zones (Fig. 4). Conversely, Nitrosopumilales, SAR202, Nitrospinales, SAR324 and UBA10353, significantly more abundant at deeper layers, exhibited a higher active/total contribution ratio in shallower layers compared to deeper layers (Fig. 4).

### Oligotypes of SAR11, Nitrosopumilales and Alteromonadales

**Figure 5:**
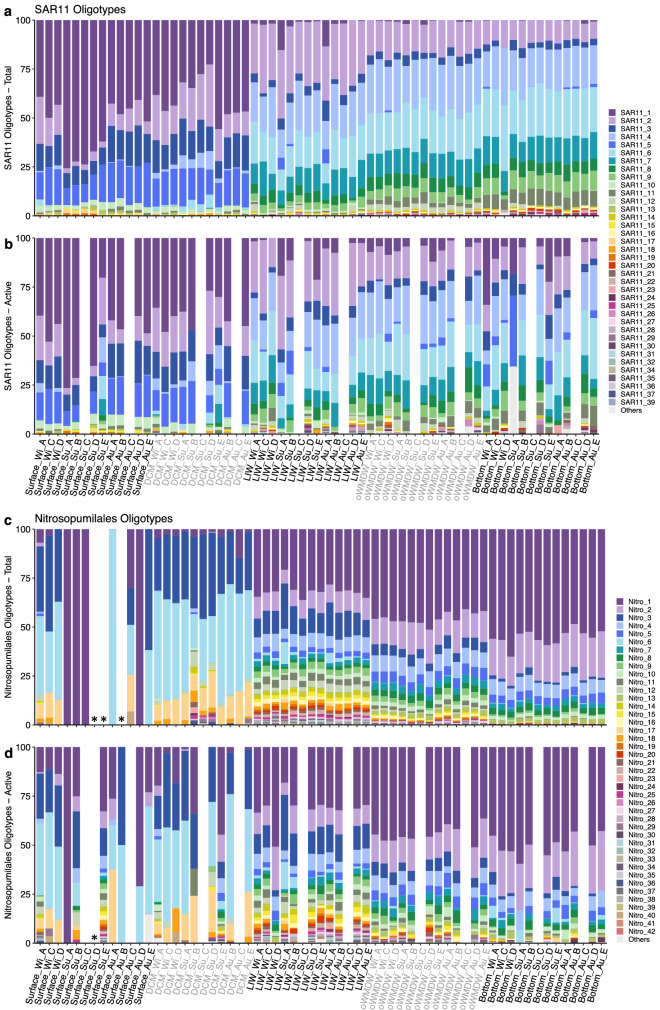
Oligotypes composition of (**a**, **b**) SAR11 and (**c**, **d**) Nitrosopumilales phylotypes for total and actively dividing samples. Stacked bar plots represent the relative abundance of oligotypes in the different samples, ordered by depth and labelled as Depth_Season_Station. Legends indicate phylotype_oligotype number. Oligotypes occurring in less than four samples are combined in ‘Others’ group. White gaps indicate samples not available or with no oligotype found (indicated by an asterisk). DCM: deep chlorophyll maximum; LIW: Levantine intermediate water; oWMDW: old western Mediterranean deep water; Wi: winter; Su: summer; Au: autumn.

Oligotyping of sequence reads from orders SAR11, Nitrosopumilales and Alteromonadales differentiated a total of 47, 50 and 44 oligotypes out of 24, 22 and 29 phylotypes at the maximum taxonomic assignment, respectively. The number of oligotypes was higher in meso- and bathypelagic compared to epipelagic layers, both of total and actively dividing communities. Nitrosopumilales oligotypes number was maximum at the LIW (Fig. 5c, d). SAR11 and Nitrosopumilales oligotype composition from total communities displayed similar patterns, with a pronounced change in oligotypes between epipelagic and meso- and bathypelagic layers (Fig. 5) and moderate contribution changes of specific oligotypes in LIW, oWMDW and bottom waters. Epipelagic and meso- and bathypelagic layers SAR11 and Nitrosopumilales oligotype composition clearly separated (Fig. 5). Redundancy analysis (RDA) with constrained environmental variables explained 89 and 86% of the variability of SAR11 and Nitrosopumilales oligotype composition, respectively (Supplementary Fig. S7). Water mass significantly explained oligotypes variation in both models (*P* = 0.001). Additionally, total vs. actively dividing communities (*P* < 0.01) significantly contributed to explain SAR11 oligotype composition; whereas nitrite (*P* < 0.05), salinity (*P* < 0.01) and oxygen (*P* = 0.001) significantly contributed to explain Nitrosopumilales oligotype composition variability (Supplementary Fig. S7 and Table S3). Pearson’s correlations revealed the co-occurrence or non-coexistence of SAR11 and Nitrosopumilales oligotypes in total communities (Supplementary Fig. S8). Particular SAR11 oligotypes were significantly positively or negatively correlated with several Nitrosopumilales oligotypes (Supplementary Fig. S8). Co-occurring oligotypes of SAR11 and Nitrosopumilales were remarkably scarcer in the actively dividing communities compared to total communities (Supplementary Fig. S8).

**Figure 6:**
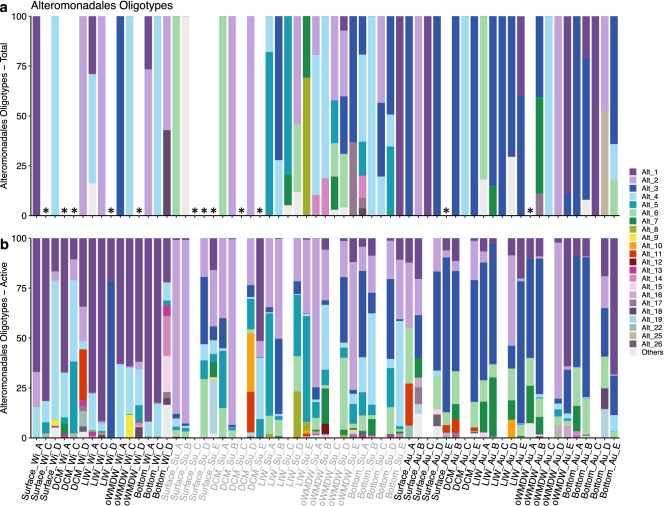
Oligotypes composition of Alteromonadales phylotype for (**a**) total and (**b**) actively dividing communities. Stacked bar plots represent the relative abundance of oligotypes in the different samples, ordered by season and labelled as Depth_Season_Station. Legend indicates phylotype_oligotype number. Oligotypes occurring in less than four samples are combined in ‘Others’ group. White gaps indicate samples not available or with no oligotype found (indicated by an asterisk). DCM: deep chlorophyll maximum; LIW: Levantine intermediate water; oWMDW: old western Mediterranean deep water; Wi: winter; Su: summer; Au: autumn.

Conversely, Alteromonadales did not show noticeable differences in oligotypes composition between water masses (Fig. 6; Supplementary Fig. S7). It should be considered that 20% of total community samples did not have enough reads assigned to Alteromonadales to efficiently define oligotypes, and, within the samples with reads, most displayed only one or two oligotypes (Fig. 6). The most abundant Alteromonadales oligotypes remarkably increased their contribution to the actively dividing communities (e.g., 1 and 2, Supplementary Fig. S9) compared to total communities. Contrarily to SAR11 and Nitrosopumilales, season (*P* = 0.001) and station (*P* = 0.001) significantly explained Alteromonadales oligotypes composition (Fig. 6; Supplementary Table S3). Several Alteromonadales oligotypes dominated in the different seasons throughout all water masses, consequently, oligotypes 1 and 4 dominated the active community during winter (Fig. 6), assigned to *Pseudoalteromonas* genus. Oligotypes 2 and 5 dominated during summer, assigned to *Alteromonas* genus; and oligotypes 3, 6 and 7 dominated during autumn (Fig. 6), the first two assigned to *Alteromonas* and the latest to *Pseudoalteromonas* genus.

## Discussion

The prokaryotic community consists of four groups: abundant but relatively less active, abundant and relatively active, rare but relatively active, and rare at both the total and the actively dividing communities. The first group significantly contributes to the community activity due to their high abundances.

SAR11, in particular Clades I and II, dominate the bulk prokaryotic community, however, they contribute relatively less to the actively dividing communities at all depth layers (Figs. 3, 4; Supplementary Fig. S6), consistent with previous studies indicating low growth rates and activity rates for this group^9,20,21,31^. SAR11 members, the most abundant organisms in the global ocean^32^, are able to oxidize a diversity of organic matter molecules as substrate, and are competitive using low-molecular-weight and labile organic compounds that are ubiquitous in oligotrophic marine environments^33,34^. The reported metabolic potential and genomic streamlining evidences the oligotrophic nature of SAR11 clade, adapted to resource limited environments^12,35^. Alternatively, the dominance of SAR11 cells in the environment was proposed to be based on SAR11 being defence specialists, resisting viral predation^22^. However, this theory is under debate since the finding of high abundances of SAR11 viruses^16^. In this study, the most abundant SAR11 phylotypes contributed relatively more to the total than to the actively dividing communities, however, they still comprised a remarkably high fraction of the active community due to their large abundance, suggesting a major role in geochemical cycles. Studies have shown different distribution and activity dynamics of SAR11 ecotypes^11,36^. Different SAR11 strains might exhibit differing ecological strategies, some being more active and highly exposed to predation pressure, whereas other strains might dominate total communities and be less active and more resistant to predation^37,38^, in agreement with the increased contribution of some SAR11 oligotypes (e.g., oligotypes 1–3, and 10) to the actively dividing communities compared to their contribution to total communities (Fig. 5; Supplementary Fig. S9). Whether different strains uphold alternative strategies^13^ or SAR11 cells spread viral immunity through recombination by DNA transfer^16^ remains under discussion.

Similar results were obtained for Nitrosopumilales, relatively abundant in the meso- and bathypelagic layers, especially at the LIW, but contributing comparatively less to the actively dividing communities (Figs. 3, 4; Supplementary Fig. S6). Lower activity rates of members of this taxa are also supported by lower 16S rRNA-rDNA ratios of OTUs affiliated to *Nitrosopumilus maritimus* (Nitrosopumilales)^39^ compared to other Thaumarchaeota taxa. However, similarly to SAR11, our results suggest that despite their lower relative activity, Nitrosopumilales might be crucial in the ocean biogeochemical cycles given their high relative abundance, especially in meso- and bathypelagic communities. It should be noted that many archaea, including the Nitrosopumilales, have autotrophic or mixotrophic metabolism^17,40^. Some autotrophic prokaryotes are not able to incorporate BrdU due to lack or inefficiency of thymidine transport systems^41–43^, and different phylotypes could differ in their capacity of BrdU assimilation^20,26^. Other constraints concerning BrdU-technique are the unlikely detection of very low abundant cells and the incubation time, i.e., longer incubation time could improve the sensitivity for slow growing cells^44^, and the potential carry-over of trace amounts of unlabelled DNA, which could result in a slight overestimation of activity of high abundance taxa (see below).

Taken together, the significant relation of Nitrosopumilales oligotypes composition with nitrite, oxygen and salinity concentrations, both in total and actively dividing communities (Supplementary Fig. S7 and Table S3), and the larger number of oligotypes differentiated at the LIW (i.e., the oxygen minimum and salinity maximum zone, Fig. 5), suggest that different Nitrosopumilales ecotypes cope with different oxygen concentrations and might exhibit different metabolic adaptations. This notion supports the reported adaptive capacity of ammonia-oxidizing archaea to different oxygen availability and its relation to nitrite and nitrous oxide production^45^.

SAR11 and Nitrosopumilales are two abundant groups, however, contributing relatively less to the actively dividing communities. Members of these two taxa did not show temporal dynamics, consistent with previous results in a nearby region^11^, however, positive and negative correlations between different oligotypes of these two taxa were determined (Supplementary Fig. S8). The co-occurrence of many SAR11 and Nitrosopumilales oligotypes was depth-related and restricted to total community, in agreement with previous findings based on OTUs^46^. This finding suggests that oligotypes of these two taxa are well adapted to similar environmental conditions. However, the lack of co-occurrence of actively dividing oligotypes might indicate that there is no direct link between the activity of the two taxa, and thus no reciprocal interactions via release/production of organic matter compounds, as indicated for other taxa^46^.

Noticeably, members of Alteromonadales are rare in the bulk prokaryotic communities (contributing 0.1% on average) but constitute a high fraction of actively dividing communities at all depth layers (contributing 28% on average), in agreement with the reported high growth rates and activities of this group^9,20,47^. Alteromonadales are considered copiotrophs^48^ with the ability to increase their growth rates in response to pulses of substrate concentrations^9,49^. However, our results show an elevated contribution to the actively dividing communities throughout all seasons and depth layers not related to increases in Chl-*a* fluorescence or nutrient concentration. Temporal variability of actively dividing Alteromonadales taxa was observed, both at family and oligotype level (Fig. 6 and Supplementary Fig. S6), indicating an intense dynamism of the rare and highly active taxa, with different oligotypes responding to seasonal and geographical changes. The seasonal change of Alteromonadales oligotypes throughout the water column could be explained by the seasonal variability of surface biotic processes, as indicated by the variation of satellite chlorophyll concentrations and particulate organic carbon (Supplementary Fig. S10), that determine the quality and quantity of organic particles from surface down to deep waters^50,51^. Alteromonadales is likely to maintain their low abundance due to grazing^23^ and/or viral lysis^22,52^ pressure^37^. The relatively high activity of Alteromonadales phylotypes together with their low abundance emphasizes their major role in dissolved organic matter degradation, especially of high-molecular weight compounds^53^, their impact on prokaryotic interactions linked to the production of extracellular enzymes^54^, and their important role in the microbial loop and viral shunt in oceanic ecosystems^52,55^.

SAR86, Actinomarinales and Rhodobacterales are also typically described as copiotrophs, able to rapidly grow and with broad metabolic potential^56,57^. Their relatively high contribution to the actively dividing communities in the epipelagic layer (Figs. 3, 4) indicates a relevant role in organic matter processing^2,58,59^, particularly in the nutrient recycling in the upper layers of oligotrophic environments such as the Mediterranean Sea.

Several phylotypes changed their relative contribution to the actively dividing communities with depth. Noteworthy, some phylotypes showed relatively high contribution to the actively dividing communities when they occurred in low abundance, whereas the same phylotypes exhibited a relatively low contribution to the actively dividing communities under conditions that triggered a high abundance in bulk communities. Synechococcales function as phototrophs in the epipelagic layer where they are abundant (Supplementary Fig. S6) and might not incorporate BrdU efficiently (Figs. 3, 4)^41,42^. However, this group could exhibit a predominantly heterotrophic lifestyle in deeper layers^60,61^, explaining the increased contribution to actively BrdU-incorporating communities in the dark ocean. Similarly, Flavobacteriales were abundant but exhibited relatively low contributions to the actively dividing communities in the epipelagic layer, whereas in the meso- and bathypelagic layers its contribution to the actively dividing community increased remarkably concurrently with a decrease in abundance. Flavobacteriales are copiotrophs related to the recycling of phytoplankton-derived organic matter and show specific bacteria-algae interactions^56^. They often dominate resource pulses following phytoplankton blooms, exhibiting high growth rates^62^. Their lower contribution to the actively dividing communities in this study coincides with oligotrophic conditions characterized by low-chlorophyll and nutrient concentrations, suggesting that Flavobacteriales phylotypes were growing slower under starving conditions in the epipelagic layer, and could become more active after a pulse of energy resources. Another potential explanation for the low contribution to actively dividing communities of Flavobacteriales would be the preferential assimilation of high vs. low molecular weight compounds^63^, such as thymidine. The higher contribution of Flavobacteriales to actively dividing communities in the meso- and bathypelagic zones could be related to a predatory role^64^ or be favoured by organic matter resuspension/release close to the bottom or sinking marine snow particles^65,66^.

The community structure of both actively dividing and total phylotypes changed in the epipelagic zone along the different seasons studied here, in agreement with previous studies of seasonal variability^39,66,67^. Bulk sunlit Mediterranean Sea prokaryotic communities exhibit drastic changes associated to seasonal variations^24^. It should be noted that the time period presented here (year 2017) coincided with a period of bulk community composition stability in the meso- and bathypelagic water masses (Supplementary Fig. S6), yet, the LIW actively dividing community changed with time, probably linked to the strong seasonal variability of the physicochemical characteristics of this water mass^68^ and the influence of particle fluxes from the sunlit ocean^69–71^.

BrdU-labelling and immunocapturing has some limitations and results have to be interpreted with caution, especially for relatively low active taxa, as a consequence of the potential presence of traces of non-labelled DNA. However, a potential overestimation of the contribution of abundant taxa to the actively dividing community would not significantly alter the result, as the abundant taxa (particularly SAR11 and Nitrosopumilales members) belong to the relatively low active community. In this study we have assessed the phylogeny of cells that were actively dividing by means of BrdU incorporation during incubations. The inter- and intra- phylotypes growth rate variations under different conditions explain the large variability of actively dividing communities’ composition, with Alteromonadales identified as key taxa that caused the largest changes in communities’ structure. The enriched BrdU-labelled community composition is consistent with results obtained with other methods to identify active taxa, such as rDNA vs. rRNA gene composition^10,72,73^, microautoradiography combined with fluorescence in situ hybridization^74,75^, proteomics^76,77^, BONCAT^78^ or other enrichment experiments^79^, indicating that the method is suitable to identify and characterize active members of the prokaryotic community. In this study it is evidenced that some low abundant phylotypes (< 0.5% abundance in total communities) are relatively active, as indicated by their increased contribution to the actively dividing communities. However, further studies are needed to characterize the factors causing the activity variability between and within phylotypes in the different environments studied.

Overall, the results presented here suggest that phylotypes that are rare or low abundant in the bulk community could be very active and dynamic not only at surface layers^80^ but also in the deep sea, with implications for the ecological interactions between microbes and the bulk community activity^81^. SAR11 and Alteromonadales, having different ecological strategies, dominate the active community throughout the water column in the western Mediterranean. Alteromonadales have been shown to produce extracellular enzymes and inhibitory compounds, whereas SAR11 scavenge the products produced by others^54,82^. Further research on potential substrates and released products by abundant and rare active phylotypes is needed to advance our understanding of the interplay between phylotypes and the structure and dynamics of active communities. Application of techniques such as sorting of BrdU-labelled cells followed by single cell genome amplification and sequencing could also further our knowledge on the metabolic potential and activity rates of the growing members of the community.

## Methods

### Field sampling and environmental parameters

Sampling was conducted at five stations in the western Mediterranean Sea in February (winter), June (summer) and November (autumn) 2017 as part of the RADMED project^83^. The stations were distributed in different regions of the western Mediterranean basin: North Balearic sub-basin (A, 41° 0′ N, 2° 37.6′ E), Mallorca Channel (B, 39° 28.6′ N, 1° 43.9′ E), South Balearic sub-basin (C, 40° 9.9′ N, 4° 36.9′ E), North Algerian sub-basin (D, 39° 0′ N, 3° 10.2′ E) and South Algerian sub-basin (E, 37° 12.3′ N, 0° 45.4′ W) (Supplementary Fig. S11). Physicochemical and bulk epi-, meso- and bathypelagic community composition characterization results have already been published in the framework of a longer time-series^24,25^.

Temperature, salinity, oxygen concentration and chlorophyll-*a* (Chl-*a*) fluorescence were recorded from surface to bottom with a SBE911 Conductivity-Temperature-Depth (CTD) sensor equipped with SBE43 and SeaPoint Fluorometer sensors. Salinity was calibrated using Guildline 8400 Autosal. Oxygen concentration was calibrated using Winkler method^84^.

Seawater samples for prokaryotic community analyses were collected at 5 depths: surface (0 m), deep chlorophyll maximum (DCM), Levantine intermediate water (LIW), old western Mediterranean deep water (oWMDW, 1000 m) and bottom water (at 5–10 m above the seafloor). The depth of the DCM and the core depth of the LIW were determined during the CTD downcast from the fluorescence profile and the T-S diagram, respectively. A total of 63 and 53 samples, respectively, were collected for bulk and actively dividing community composition analysis: 24 samples for each depth except the DCM with 20. Additional samples for inorganic nutrients concentration and prokaryotic abundance were taken at 25, 50, 75, 100, 200, 300, 700 and 1500 m.

12 mL seawater were collected and stored frozen at − 20 °C for dissolved inorganic nutrients analysis. Nitrate (NO_3_^−^), nitrite (NO_2_^−^), phosphate (PO_4_^3−^) and silicate (SiO_4_^2−^) concentrations were determined using a QuAAtro Gas Segmented Continuous Flow Analyser (SEAL Analytical) following colorimetric methods^84–86^.

### Prokaryotic abundance

1.5 mL seawater was fixed with glutaraldehyde (0.1% final concentration), frozen in liquid nitrogen and stored at − 80 °C until further processing. Prokaryotic abundance was determined by flow cytometry using an ACCURI C6 (BD Biosciences) instrument. Samples were thawed, stained with SYBR Green I (Sigma-Aldrich, 1 × final concentration) for 10 min in the dark and counted based on their side scatter versus green fluorescence signatures in a cytogram^87^. Fluorescent calibration beads (Fluospheres polystyrene 1.0 µm, Molecular probes) were added as internal standard. Based on the relative green fluorescence signal, two populations were distinguished by gating: high nucleic acid (HNA) and low nucleic acid (LNA) cells^88^.

### Bulk and actively dividing prokaryotic community DNA

4L of seawater were filtered onto 0.2 μm polycarbonate filters (47 mm diameter, Whatman, Nucleopore) for total prokaryotic community composition assessment. The actively dividing community was determined based on BrdU labelling and immunocapturing^20^. 4L of seawater were incubated 24 h with BrdU (20 nM final concentration) at in situ temperature in the dark. Subsequently, the water was filtered onto 0.2 μm polycarbonate filters. Following, filters from bulk and BrdU-labelled samples were flash-frozen in liquid nitrogen and stored at − 80 °C until DNA extraction.

DNA was extracted as previously described^24^. Briefly, filters were cut into small pieces with sterile scissors and incubated 45 min with lysozyme at 37 °C followed by 1 h with proteinase K at 55 °C for enzymatic lysis. Zirconium beads were added for mechanic lysis during 10 min followed by 30 min incubation at 70 °C. DNA was extracted and purified sequentially with phenol (pH 8), phenol:chloroform:isoamyl alcohol (25:24:1) and chloroform. DNA was precipitated with 0.02 volumes of 5 M NaCl and 2 volumes of cold ethanol at − 20 °C, recovered by centrifugation (21,000×*g*) and washed with ice cold 70% ethanol. DNA was stored at − 80 °C resuspended in sterile DNAse/RNAse free water.

### BrdU immunocapture

BrdU-labelled DNA was isolated by magnetic bead immunocapture, following Hamasaki et al.^20^ with some modifications. All incubations were performed at room temperature. Herring sperm DNA (1.25 mg mL^−1^ in PBS; Invitrogen) was boiled for 1 min and immediately frozen in dry-ice ethanol. Once thawed, the herring sperm DNA was mixed (9:1) with anti-BrdU monoclonal antibody (diluted 1:10 in PBS; Sigma-Aldrich) and incubated 30 min. DNA extracts from BrdU-labelled samples were boiled for 1 min and immediately frozen in dry-ice ethanol. Once thawed, the denatured DNA was mixed with 10 µL of the herring sperm DNA-antibody mixture and incubated for 30 min.

Paramagnetic beads (Dynabeads Goat anti-Mouse IgG, Invitrogen) were washed two times with PBS containing BSA (acetylated albumin from bovine serum; Sigma-Aldrich) (0.1 mg mL^−1^) using a magnetic concentrator. The beads were resuspended in PBS-BSA at their initial concentration. 25 µL of beads suspension were added to 20 µL of sample DNA mixture and incubated 30 min in constant agitation. Beads-DNA mixture was then washed seven times with 0.5 mL PBS-BSA, and kept on constant agitation for 10 min between each wash. The washed beads attached to BrdU-labelled DNA were resuspended in 20 µL of DNAse/RNAse free water and stored at − 80 °C. DNA samples without BrdU labelling corresponding to samples from the same locations and depths were processed in the same manner and subjected to PCR as negative control. BrdU-labelled samples always showed more pronounced amplification than non-labelled samples, however it cannot be excluded that traces of unlabelled DNA are carried with the beads. Thus, we analysed here the enriched BrdU-labelled community, subsequently referred to as actively dividing community for simplicity. It has to be noted that potential traces of unlabelled DNA could mainly bias the composition of the low active cells, potentially overestimating the contribution to the actively dividing community of taxa that is abundant in the total community.

### 16S rRNA gene amplification and sequencing

The 16S rRNA gene was amplified for both total and BrdU-labelled communities. The primers used for amplification were 515F-Y (5′-GTGYCAGCMGCCGCGGTAA) and 926R (5′-CCGYCAATTYMTTTRAGTTT), targeting the hypervariable regions V4 and V5 of the 16S rRNA genes of Archaea and Bacteria and the eukaryotic 18S rRNA gene^89^. Reaction mixtures for amplification contained 1 µL DNA extract, primers (1 µM final concentration) and 1 × Kapa HiFi HotStart ReadyMix (Kapa Biosystems). Cycling conditions followed Parada et al.^89^. PCR products were purified using the Qiaquick PCR Purification Kit (Qiagen), according to manufacturer’s instructions. Amplified 16S rRNA gene fragments were checked by electrophoresis on 2% agarose gels.

Eukaryotic 18S rRNA was significantly amplified in BrdU-labelled communities. 18S rRNA gene amplicons were removed from BrdU-labelled samples by gel extraction and purification using Qiaquick Gel Extraction Kit (Qiagen), following manufacturer’s protocol, since this study focused on archaea and bacteria. Purified 16S rRNA gene amplicons of both total and BrdU-labelled communities were sequenced using MiSeq Illumina 2 × 250 bp.

### Bioinformatics and statistics

Bioinformatic analysis of 16S rRNA gene followed the pipeline implemented in QIIME2 (http://qiime2.org)^90^. Sequences were demultiplexed and quality filtered using DADA2^91^. The denoiser algorithm implemented in QIIME2 was used to remove sequencing errors and chimeras. Sequences with ambiguities were removed (maxN = 0), the length of forward and reverse reads was truncated to 245 bp and 230 bp, respectively, and the primers length was trimmed (trimLeft). Sequences assigned to ‘Chloroplasts’ were removed for further analyses. A total number of 3,199,836 sequences were retained for further analyses after filtering. MAFFT^92^ pipeline was used for Amplicon Sequence Variants (ASVs) alignment. Phylogenetic trees were constructed using IQ-TREE (www.iqtree.org) and midpoint rooting algorithms. Alpha and beta diversity estimates were assessed with the ASVs table rarefied to 4,729 sequences. Taxonomy was assigned using SILVA classifier (release 132).

Oligotyping analysis^93^ was conducted on SAR11, Nitrosopumilales and Alteromonadales lineages as described in http://oligotyping.org pipeline. A total of 9, 6 and 9 highly variable base positions were used to assess the oligotypes for SAR11, Nitrosopumilales and Alteromonadales, respectively, based on entropy. Oligotypes that occurred in more than 0.5% of reads (a = 0.5) were considered. Pearson’s correlation was used to identify co-occurrence patterns between SAR11 and Nitrosopumilales oligotypes, distinguishing between total and actively dividing populations. Taxonomy at genus level was assigned for Alteromonadales oligotypes using BLAST (http://blast.ncbi.nlm.nih.gov) based on maximum scores (top hits with assignment consistency, 100% identity and 100% sequence cover).

Analysis of variance (ANOVA) and permutational multivariate analysis of variance (PERMANOVA) were used to determine significant depth-related differences between diversity measures and the statistically significant variables explaining community variance based on Bray–Curtis, Jaccard, weighted and unweighted UniFrac distance matrices, respectively. Only non-colinear variables were used, selected through Variation Inflation Factor (VIF) test. The analyses were performed using the ‘aov’ and ‘adonis’ functions of the ‘vegan’ package of R, using a significance level of *P* < 0.05 based on 999 permutations. Principal Coordinates Analysis (PCoA) was used to visualize community variance in a two-dimensional ordination according to the distance matrices previously calculated. The active to total ratio of a phylotype was calculated by dividing its contribution to the actively dividing community by its contribution to the total community. Redundancy analysis (RDA) was used to relate oligotypes composition with environmental parameters using the ‘rda’ function of the ‘vegan’ package of R. Only non-collinear variables were used for the RDA models.

## Supplementary Information

Supplementary Information.

## Data Availability

16S rRNA raw sequence data has been deposited in NCBI sequence read archive (SRA) under the accession numbers PRJNA612168, PRJNA575848 and PRJNA638520.
