# Supplementary material for: Dynamics of actively dividing prokaryotes in the western Mediterranean Sea
Source: Sci Rep. 2022 Feb 8;12:2064. doi: 10.1038/s41598-022-06120-y (PMC8825817; doi:10.1038/s41598-022-06120-y)
Supplement: Supplementary file 1 — Supplementary Information. [file 41598_2022_6120_MOESM1_ESM.pdf]

SUPPLEMENTARY INFORMATION (SI)

SCIENTIFIC REPORTS

Dynamics of actively dividing prokaryotes in the western Mediterranean Sea

Catalina Mena*, Patricia Reglero, Rosa Balbín, Melissa Martín, Rocío Santiago, Eva Sintes

*Corresponding author: Catalina Mena, Catalina.Mena.Oliver@ifremer.fr. Current affiliation and address: IFREMER – Centre Bretagne Z.I. Technopôle Brest-Iroise Pointe du Diable BP70 29280 Plouzané, France.

Supplementary Figure S1

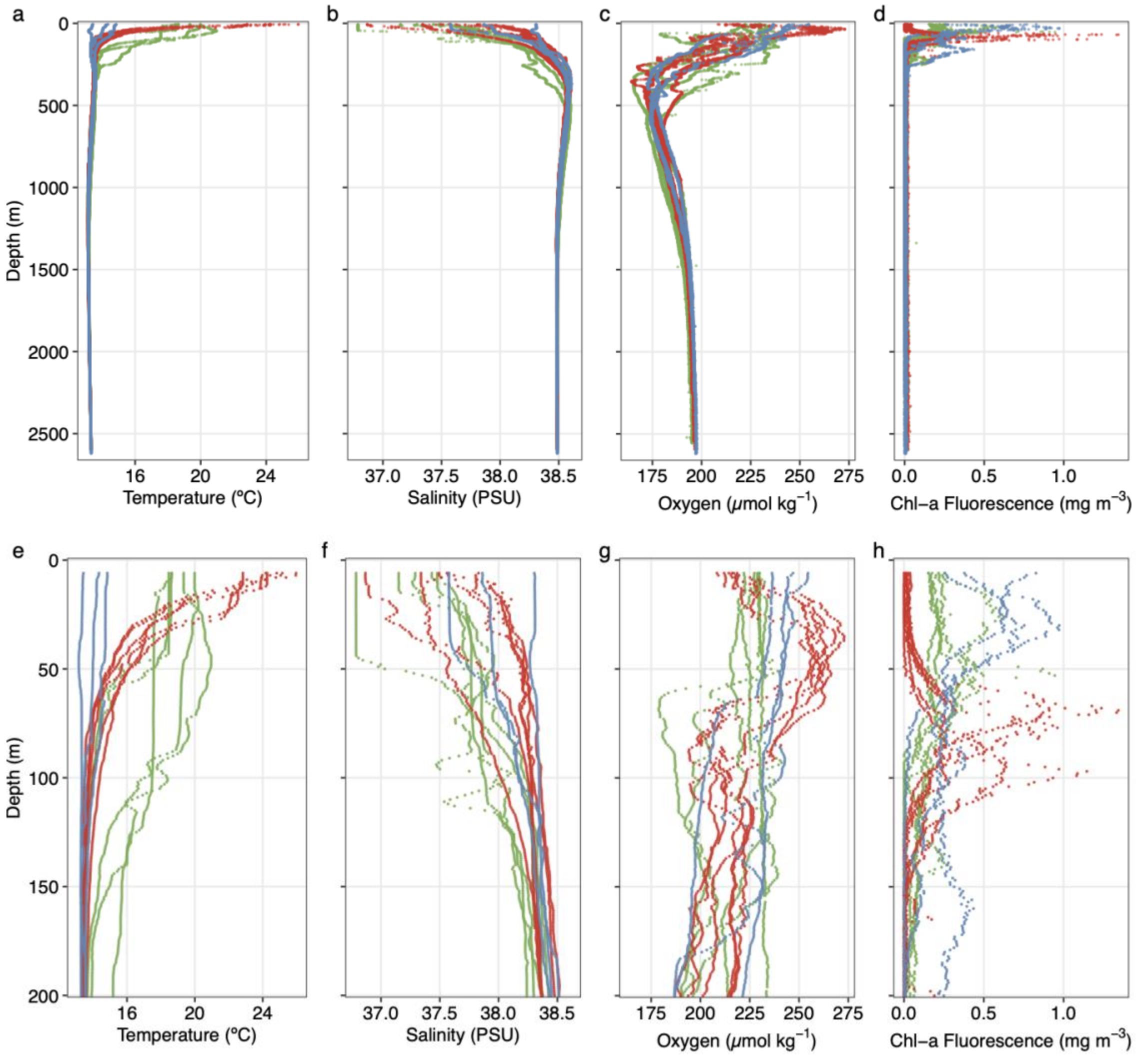

Figure S1. Profiles of temperature (a, e), salinity (b, f), oxygen (c, g) and chlorophyll-*a* fluorescence (d, h) of all sampled stations. Panels a-d show data from surface to bottom, panels e-h show same data zoomed from surface to 200 m. Colours indicate season: blue for winter (February 2017), red for summer (June 2017) and green for autumn (November 2017).

Supplementary Figure S2

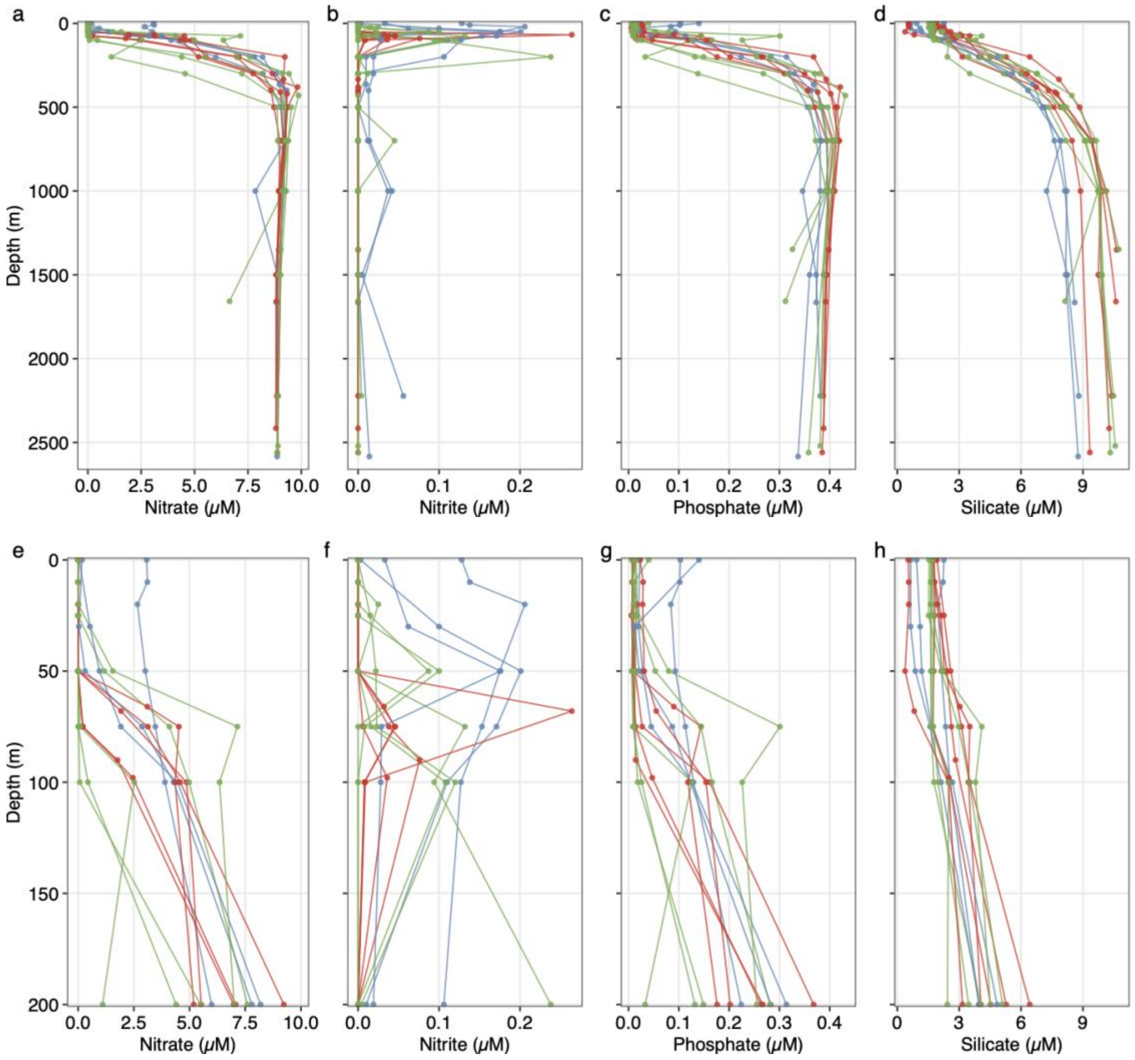

Figure S2. Depth profiles of inorganic nutrients concentrations: (a, e) nitrate, (b, f) nitrite, (c, g) phosphate and (d, h) silicate. Panels a-d show data from surface to bottom, panels e-h show same data for the upper 200 m. Colours indicate season: blue for winter (February 2017), red for summer (June 2017) and green for autumn (November 2017).

Supplementary Figure S3

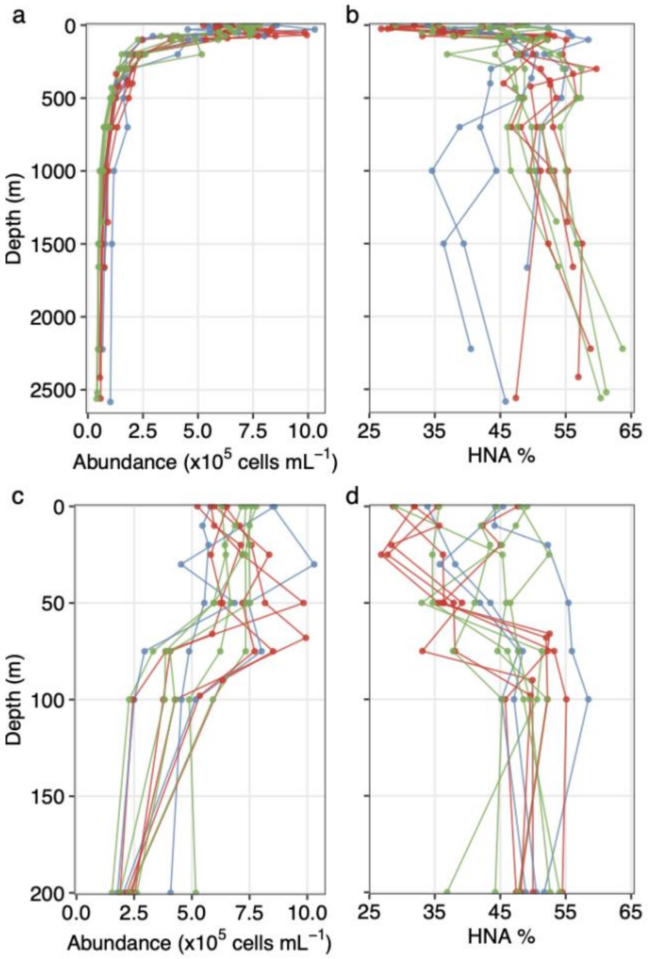

Figure S3. Depth profiles of (a, c) total prokaryotic abundance and (b, d) HNA relative abundance. Panels a and b show data from surface to bottom, panels c and d show same data for the upper 200 m. Colours indicate season: blue for winter (February 2017), red for summer (June 2017) and green for autumn (November 2017).

Supplementary Figure S4

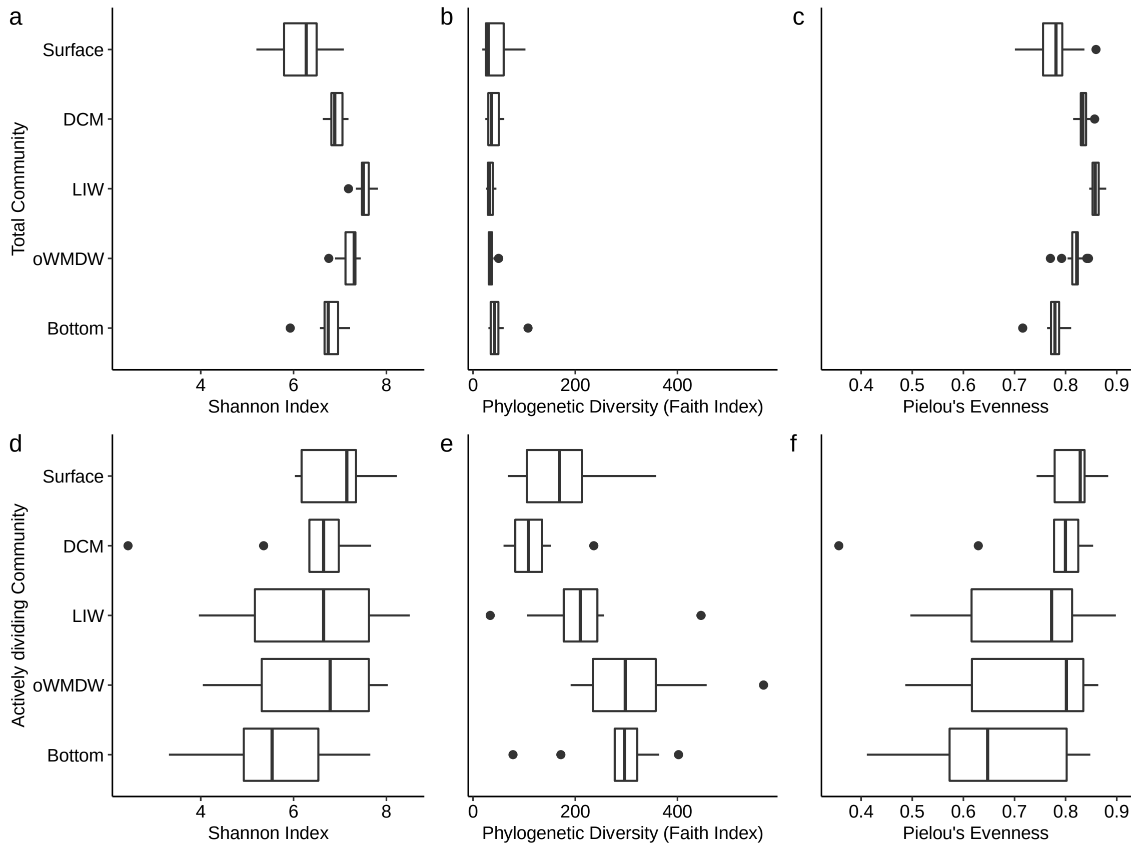

Figure S4. Alpha diversity indexes at different depth layers for total (a-c) and actively dividing (d-f) communities. Boxplots show the median (dark vertical line inside the boxes), the first and third quartile (boxes) and the variability outside the first and third quartile (extending lines). Black dots indicate outliers and grey diamonds indicate jitter values. DCM: deep chlorophyll maximum; LIW: levantine intermediate water; oWMDW: old western Mediterranean deep water.

Supplementary Figure S5

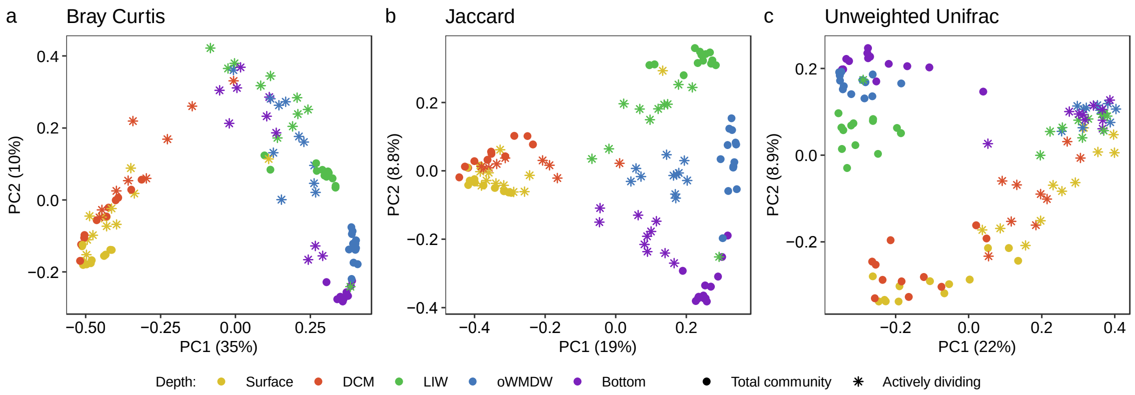

Figure S5. Principal coordinates analysis (PCoA) based on (a) Bray Curtis, (b) Jaccard and (c) unweighted UniFrac distances between the prokaryotic communities. The proportion of variance explained is shown for each axis. Colours indicate water layers and shapes indicate total versus actively dividing communities. DCM: deep chlorophyll maximum; LIW: levantine intermediate water; oWMDW: old western Mediterranean deep water.

Supplementary Figure S6

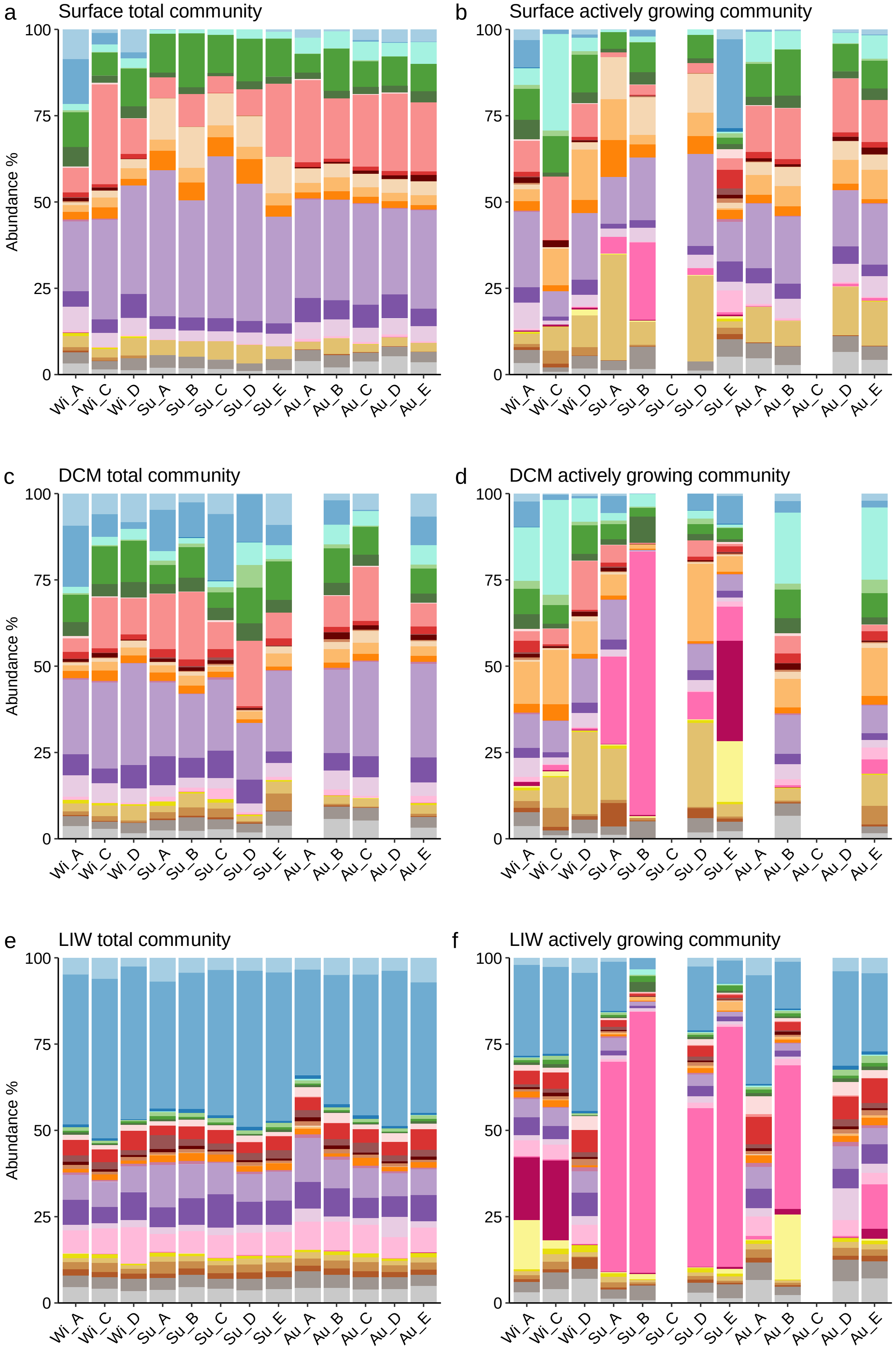

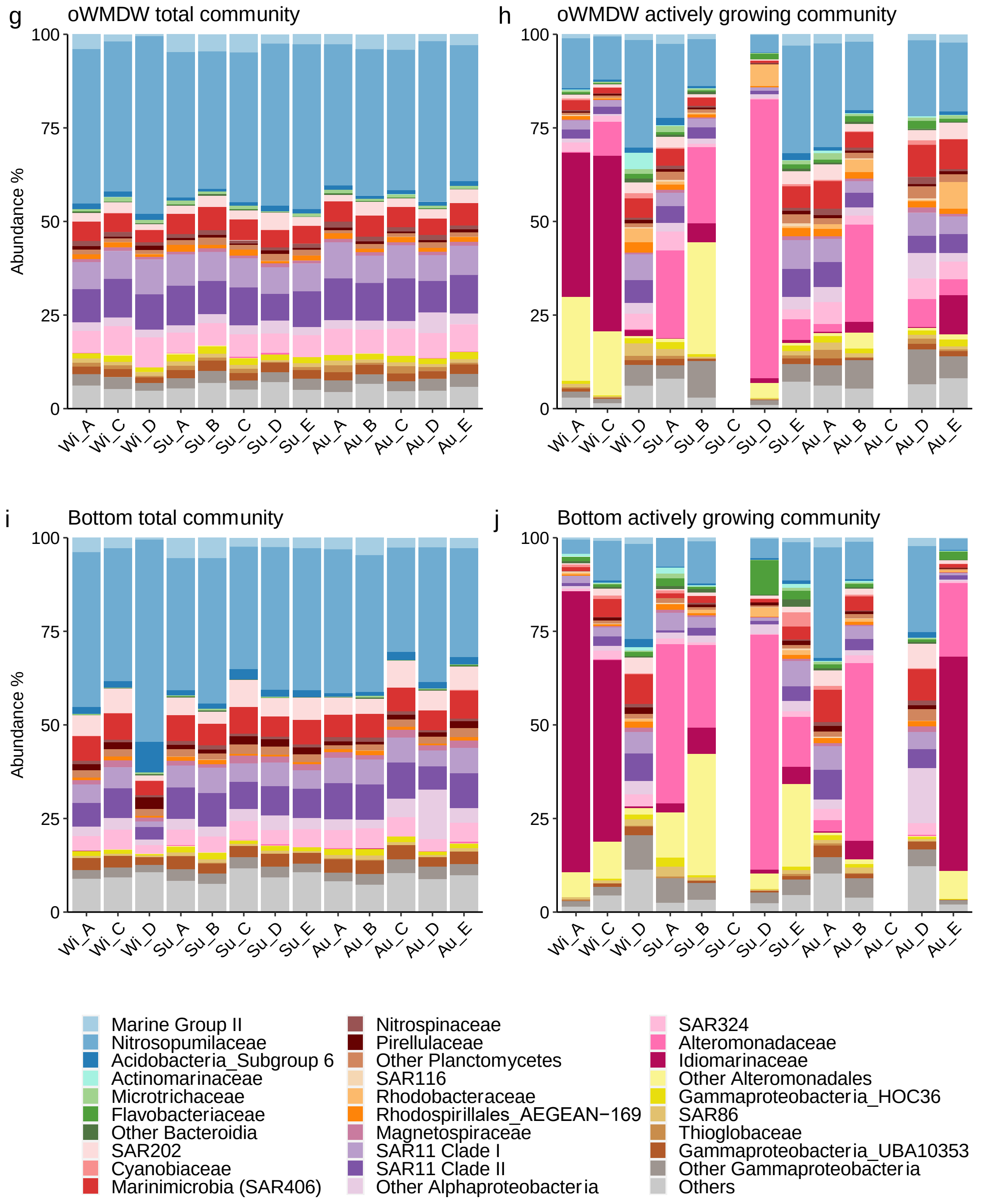

Figure S6. Prokaryotic community composition of (a, c, e, g, i) total and (b, d, f, h, j) actively dividing communities in the different depth layers. Stacked bar plots represent the relative abundance of phylotypes at the family level at each sampled station, labelled with Season_Station (Wi: winter; Su: summer; Au: autumn). Phylotypes contributing <0.5% throughout the study are combined in 'Others' group. White gaps indicate no sampling: in case of DCM of St. A and D in autumn because the a DCM was not detected and the gaps in actively growing communities because BrdU enrichment was not done.

DCM: deep chlorophyll maximum; LIW: levantine intermediate water; oWMDW: old western Mediterranean deep water.

Supplementary Figure S7

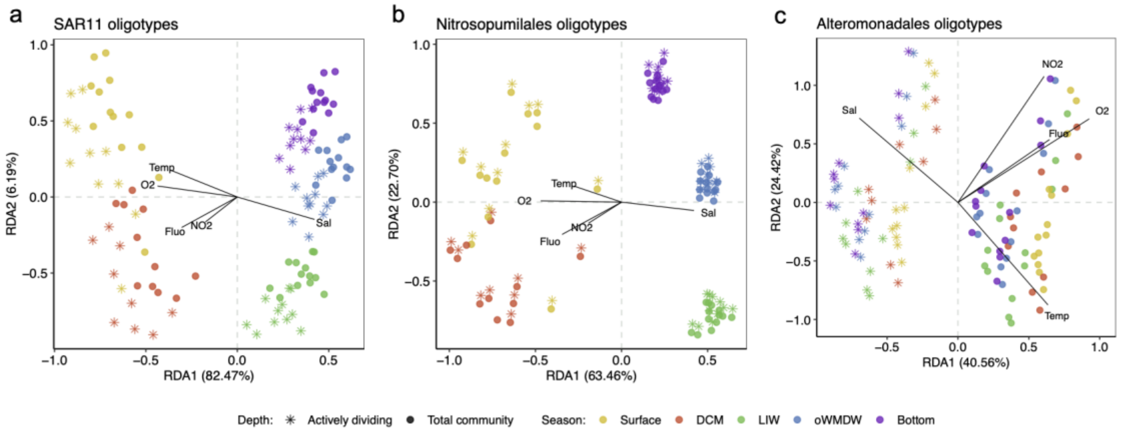

Figure S7. Redundancy analysis (RDA) for (a) SAR11, (b) Nitrosopumilales and (c) Alteromonadales oligotypes composition with constraint variables. The percentage of variance explained is shown for each axis. Vectors represent the constrained continuous variables used in the RDA models. Strength of relative correlations with RDA axes is indicated by the length and direction of vectors. Colours indicate water layers and shapes indicate total vs. actively dividing communities. Results of the models are shown in Supplementary Table S4. Fluo: Chlorophyll-*a* fluorescence; NO2: nitrite concentration; O2: dissolved oxygen; Sal: salinity; Temp: potential temperature. DCM: deep chlorophyll maximum; LIW: levantine intermediate water; oWMDW: old western Mediterranean deep water.

Supplementary Figure S8

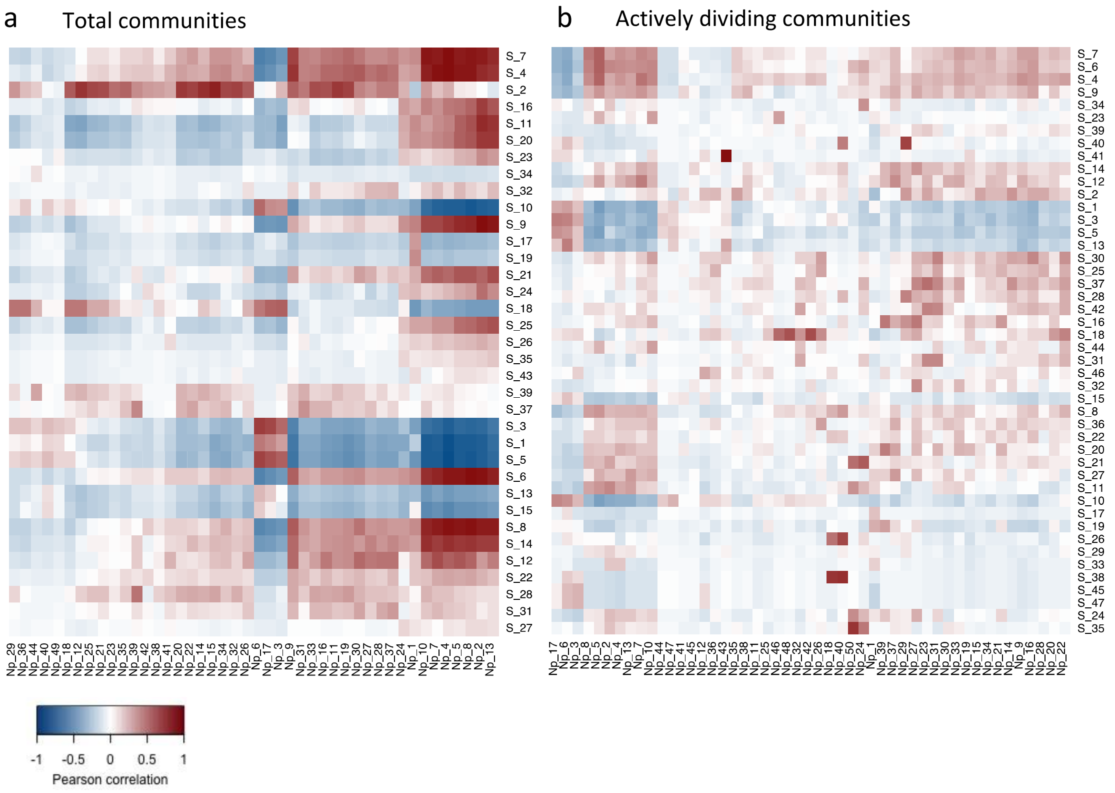

Figure S8. Pearson correlations between SAR11 (y axis) and Nitrosopumilales (x axis) relative abundance oligotypes for (a) total and (b) actively dividing communities. Red indicate positive correlation (i.e., co-occurrence) and blue indicate negative correlation (i.e., non-coexistence).

Supplementary Figure S9

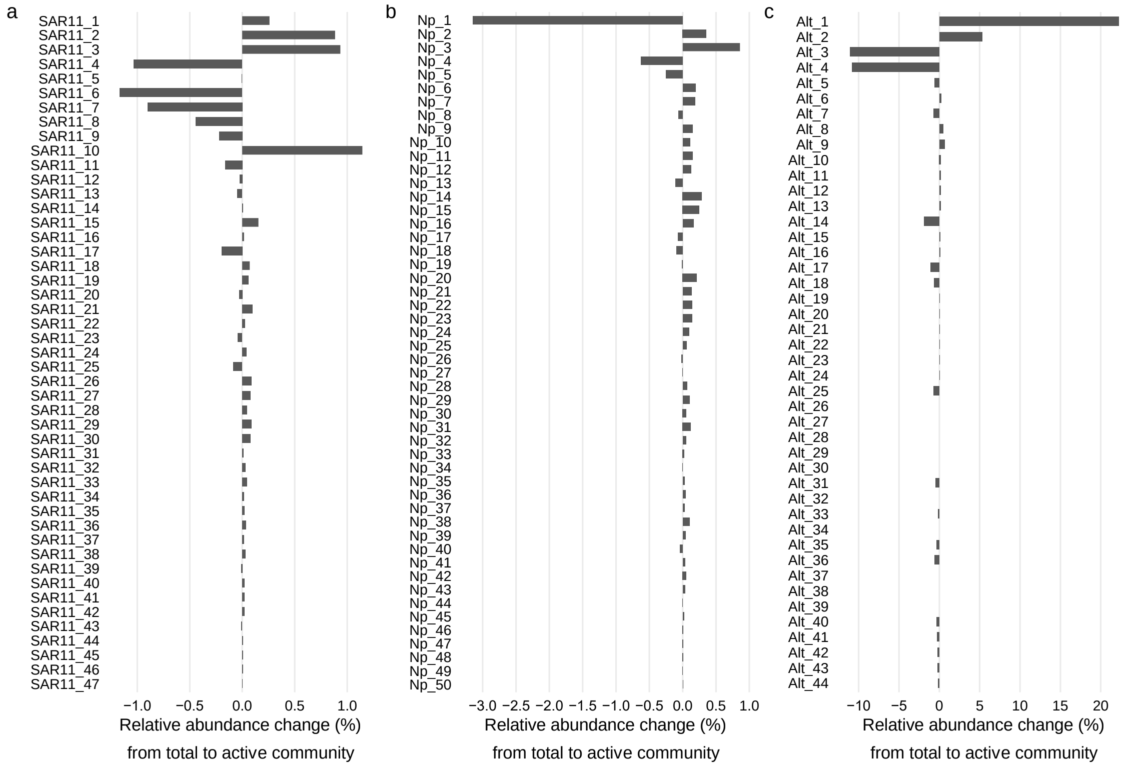

Figure S9. Relative abundance changes (%) from total to actively dividing communities of (a) SAR11, (b) Nitrosopumilales and (c) Alteromonadales oligotypes. Positive values indicate the % increase in actively dividing compared to total communities and negative values the % increase in total compared to actively dividing communities. Y axis indicate phylotype_oligotype number. Np: Nitrosopumilales; Alt: Alteromonadales.

Supplementary Figure S10

Winter (2 to 9 Feb 2017)

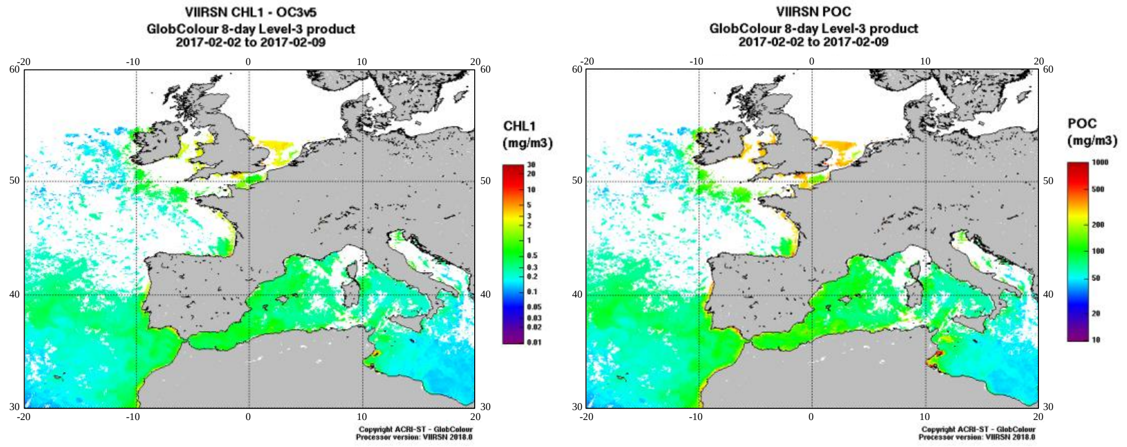

Summer (10 to 17 Jun 2017)

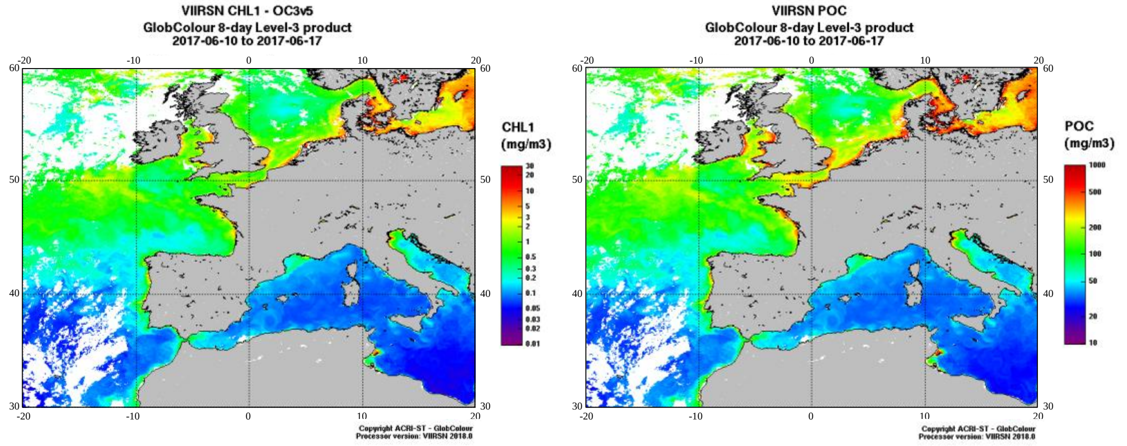

Autumn (17 to 24 Nov 2017)

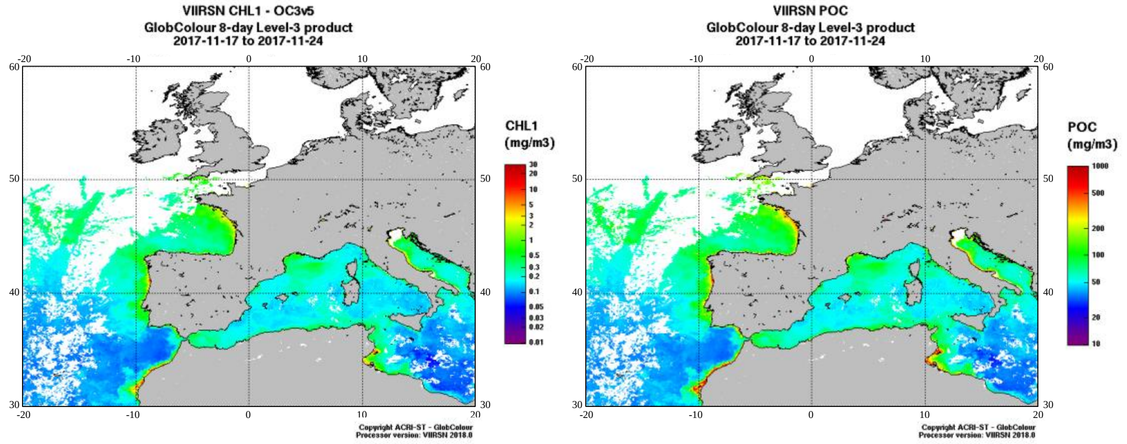

Figure S10. Maps of the satellite derived chlorophyll *a* concentration (CHL, mg m^{-3}) and particulate organic matter (POC, mg m^{-3}) in the western Mediterranean basin for the three seasons studied. Weekly satellite data products were obtained from the GlobColour project (http://www.globcolour.info/).

Supplementary Figure 11

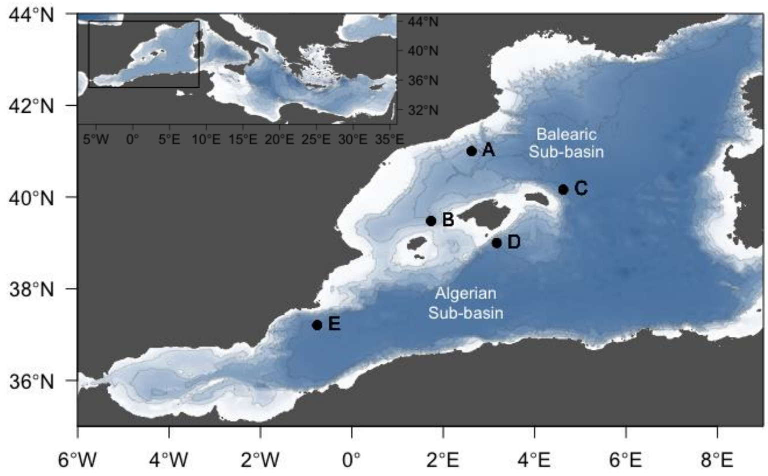

Figure S11. Location of sampling stations (labelled black dots) in the western basin of the Mediterranean Sea. The bottom depth at station A: 1670 m, B: 1370 m, C: 2500 m, D: 2300 m and E: 2560 m.

Supplementary Table S1. PERMANOVA (nonparametric ANOVA) results based on weighted UniFrac, Bray Curtis, Jaccard and unweighted UniFrac distances of prokaryotic communities, showing the significance of explanatory variables using a permutation test (999 permutations). The variable ‘activity’ denotes total vs. actively dividing communities. ‘P. temperature’ indicates potential temperature. The sum of squares, R^2 and probability (P-value) are shown. Number of asterisks indicate the significance level of each variable (*** P <0.001, ** P <0.01, * P <0.05, no asterisk = P >0.05). The function ‘adonis’ from the R package was used for the calculations. Silicate, phosphate and nitrate concentrations had been excluded from the analyses due to collinearity (using VIF test in R). PCoA plots for the four distance matrices are shown in Fig. 1 in the manuscript and Fig. S5 in the Supplementary Information.

**Variable**	**Sum of Sqs**	**R^2**	**P-value**
Weighted UniFrac			
Water mass	13.781	0.27854	0.001***
Activity	12.922	0.26117	0.001***
Season	0.946	0.01912	0.031*
Station	0.764	0.01545	0.507
Oxygen (µmol kg^{-1})	0.340	0.00687	0.158
Nitrite (µM)	0.254	0.00513	0.256
Salinity	0.433	0.00875	0.097
Chl-*a* (mg m^{-3})	0.107	0.00217	0.639
P. Temperature (ºC)	0.118	0.00238	0.626
Residuals	*19.811*	*0.40041*	-
Bray Curtis			
Water mass	16.725	0.42935	0.001***
Activity	2.640	0.06776	0.001***
Season	1.353	0.03474	0.001***
Station	0.910	0.02335	0.085
Oxygen (µmol kg^{-1})	0.871	0.02236	0.001***
Nitrite (µM)	0.232	0.00596	0.146
Salinity	0.405	0.01039	0.017*
Chl-*a* (mg m^{-3})	0.355	0.00912	0.034*
P. Temperature (ºC)	0.336	0.00862	0.039*
Residuals	*15.128*	*0.38834*	-
Jaccard			
Water mass	12.628	0.28067	0.001***
Activity	1.265	0.02812	0.001***
Season	1.236	0.02747	0.001***

Station	1.265	0.02811	0.122
Oxygen (µmol kg^{-1})	0.817	0.01816	0.002**
Nitrite (µM)	0.328	0.00729	0.158
Salinity	0.484	0.01076	0.019*
Chl-*a* (mg m^{-3})	0.461	0.01025	0.030*
P. Temperature (ºC)	0.468	0.01041	0.025*
Residuals	*26.039*	*0.57875*	-
Unweighted UniFrac			
Water mass	4.379	0.11364	0.001***
Activity	7.001	0.18168	0.001***
Season	0.692	0.01796	0.054
Station	0.992	0.02573	0.381
Oxygen (µmol kg^{-1})	0.530	0.01374	0.008**
Nitrite (µM)	0.247	0.00642	0.349
Salinity	0.332	0.00862	0.114
Chl-*a* (mg m^{-3})	0.367	0.00953	0.083
P. Temperature (ºC)	0.244	0.00632	0.375
Residuals	*23.750*	*0.61636*	-

Supplementary Table S2. PERMANOVA (nonparametric ANOVA) results based on weighted UniFrac distances of prokaryotic communities computed for the different depth layers separately. The results show the significance of explanatory variables using a permutation test (999 permutations). Only non-collinear variables were used for each analysis. The variable 'activity' denotes total vs. actively dividing communities. 'P. temperature' indicates potential temperature. The interaction of two variables is indicated by an asterisk between them. The sum of squares, R^2 and probability (P-value) are shown. Number of asterisks indicate the significance level of each variable (*** P <0.001, ** P <0.01, * P <0.05, no asterisk = P >0.05). The function 'adonis' from the R package was used for the calculations. Depth layers: Surface; DCM, deep chlorophyll maximum; LIW, levantine intermediate water; oWMDW, old western Mediterranean deep water and bottom.

**Variable**	**Sum of Sqs**	**R^2**	**P-value**
Surface			
Activity	0.94181	0.30412	0.001***
Season	0.59296	0.19147	0.006**
Station	0.36966	0.11937	0.257

Salinity	0.15296	0.04939	0.097
Silicate (µM)	0.03553	0.01147	0.763
Phosphate (µM)	0.05886	0.01901	0.554
Oxygen (µmol kg^{-1})	0.04020	0.01298	0.694
Residuals	*0.90485*	*0.29219*	-
DCM			
Activity	1.06133	0.42233	0.001***
Season	0.24817	0.09875	0.161
Station	0.28245	0.11240	0.567
Salinity	0.11632	0.04629	0.208
Phosphate (µM)	0.04061	0.01616	0.764
Nitrite (µM)	0.05608	0.02232	0.572
Oxygen (µmol kg^{-1})	0.07873	0.03133	0.383
Residuals	*0.62933*	*0.25043*	-
LIW			
Activity	2.7963	0.48808	0.001***
Season	0.5609	0.09701	0.066
Activity*Season	0.5988	0.10451	0.047*
Station	0.4040	0.07052	0.457
Oxygen (µmol kg^{-1})	0.1246	0.02174	0.311
Nitrite (µM)	0.1376	0.02401	0.274
Salinity	0.0559	0.00975	0.607
P. Temperature (ºC)	0.0331	0.00577	0.732
Residuals	*0.14804*	*0.17771*	-
oWMDW			
Activity	5.8032	0.36415	0.001***
Season	1.2585	0.07897	0.313
Station	1.2525	0.07859	0.816
Nitrite (µM)	0.2847	0.01787	0.582
Nitrate (µM)	0.3202	0.02009	0.541
Salinity	0.2912	0.01827	0.511
Residuals	*0.5174*	*0.42205*	-
Bottom			
Activity	5.1573	0.61252	0.001***
Season	0.2010	0.02387	0.734
Station	0.5905	0.07014	0.616
Nitrite (µM)	0.2831	0.03363	0.236
Phosphate (µM)	0.0374	0.00444	0.863
Nitrate (µM)	0.0747	0.00887	0.683
Salinity	0.0640	0.00760	0.740
Oxygen (µmol kg^{-1})	0.0487	0.00578	0.807
Residuals	*1.9631*	*0.23315*	-

Supplementary Table S3. Results of the redundancy analysis (RDA) for SAR11, Nitrosopumilales and Alteromonadales oligotypes composition using 999 permutations. Only non-collinear variables were used in the models, which include station, water mass, season, activity, nitrite concentration, salinity, dissolved oxygen, Chl-*a* fluorescence (Fluo) and potential temperature (P Temp). The variable 'activity' denotes total vs. actively dividing communities. The estimated degrees of freedom (Df), variability (Var), F statistic value and probability (P-value) are indicated for the three models, for the first and second RDA axes and for the constrained variables. Number of asterisks indicate the significance level of each variable (*** P <0.001, ** P <0.01, * P <0.05, no asterisk = P >0.05). The function 'rda' from the R package was used for the calculations.

	SAR11 oligotypes				*Nitrosopumilales oligotypes*			
	**Df**	**Var**	**F**	**P value**	**Df**	**Var**	**F**	**P value**
**Model**	16	5.4298	11.828	0.001***	16	7.6264	13.17	0.001***
Residual	*99*	*2.8404*			*99*	*3.5829*		
R^2 *adj*	*0.60*				*0.63*			
**Axes**								
RDA1	1	4.4779	156.074	0.001***	1	4.8400	133.7338	0.001***
RDA2	1	0.3362	11.7171	0.001***	1	1.7309	47.8279	0.001***
**Constrained variables**								
Station	4	0.1399	1.1672	0.273	4	0.2072	1.4314	0.115
Water mass	4	4.7745	41.6026	0.001***	4	6.4557	44.5942	0.001***
Season	2	0.0924	1.6104	0.164	2	0.1740	2.4036	0.024*
Activity	1	0.1590	5.5403	0.007**	1	0.0759	2.0969	0.070
Nitrite	1	0.0345	1.2032	0.242	1	0.1004	2.7729	0.031*
Salinity	1	0.0842	2.9362	0.040*	1	0.1779	4.9159	0.002**
Oxygen	1	0.0617	2.1504	0.092	1	0.2440	6.7414	0.001***
Fluo	1	0.0483	1.6833	0.152	1	0.1236	3.4160	0.019*
P Temp	1	0.0412	1.4369	0.193	1	0.0678	1.8728	0.093
Residual	*99*	*2.8404*			*99*	*3.5829*		

Alteromonadales oligotypes				
	**Df**	**Var**	**F**	**P value**
**Model**	16	3.8995	3.9942	0.001***
Residual	*99*	*6.0408*		
R^2 *adj*	*0.29*			
**Axes**				
RDA1	1	1.5814	25.9174	0.001***
RDA2	1	0.9522	15.6056	0.001***
**Constrained variables**				
Station	4	0.6765	2.7717	0.001***
Water mass	4	0.4461	1.8276	0.006**
Season	2	1.1674	9.5657	0.001***
Activity	1	1.3459	22.0579	0.001***
Nitrite	1	0.0500	0.8201	0.587
Salinity	1	0.0652	1.0689	0.385
Oxygen	1	0.0489	0.8013	0.595
Fluo	1	0.0707	1.1592	0.320
P Temp	1	0.0287	0.4710	0.921
Residual	*99*	*6.0408*		
